# Supplementary material for: Are carbon emissions trading and green financial instruments synergistic? -Comprehensive quantitative research based on content analysis
Source: PLoS One. 2024 Mar 7;19(3):e0298601. doi: 10.1371/journal.pone.0298601 (PMC10919601; doi:10.1371/journal.pone.0298601)
Supplement: S1 Data — (ZIP) [file pone.0298601.s001.zip › Data/地级市Z_F工作报告.zip!/░▓╗╒/╬▀║■╩╨/└┤╫╘╣1⁄2╓┌║┼í╛┬φ┐╦╩2╛▌═°í┐_2010.docx]

——2010年1月12日在芜湖市第十四届人民代表大会第四次会议上

市长 杨敬农

各位代表：

现在，我代表市人民政府，向大会作政府工作报告，请予审议，并请市政协各位委员和其他列席人员提出意见。

一、2009年工作回顾

刚刚过去的2009年，是我市发展形势极为复杂、极为严峻的一年，也是全市人民团结一心、共克时艰的一年。一年来，在中共芜湖市委的坚强领导下，全市上下深入学习实践科学发展观，紧紧围绕保增长、保民生、保稳定，同心同德，砥砺奋进，较好地完成了市十四届人大三次会议确定的目标任务，经济社会呈现出增长较快、结构优化、后劲增强、民生改善、社会和谐的良好态势。

初步统计，全市实现生产总值900亿元，比上年增长16.2%；规模以上工业实现增加值463亿元，增长25%；全社会固定资产投资892亿元，增长45%；社会消费品零售总额241亿元，增长18.8%；财政收入147.8亿元，增长19.3%，其中地方财政收入69.4亿元，增长27.6%；城市居民人均可支配收入16700元，增长12%；农民人均纯收入6690元，增长9%；城镇新增就业岗位4.8万个，城镇登记失业率控制在4%以内；人口出生率7.32‰。完成了省政府下达的节能减排任务。

一年来，我们主要做了六个方面的工作：

（一）全力保持经济平稳较快增长。面对国际金融危机严重冲击，我们准确把握形势，果断决策，主动作为。一是强化战略谋划。牢牢把握发展这个第一要务，坚定信心不动摇，提出了GDP人均赶合肥、总量超湖州的奋斗目标。抢抓国家设立皖江城市带承接产业转移示范区的重大历史机遇，积极对接争取，芜湖核心城市的战略定位、重大基础设施项目、重要产业布局以及更加优惠的发展政策都被纳入初步规划，为芜湖当前及长远发展赢得了难得的有利条件。及时出台了推进工业强市、三产兴市、城乡统筹的一系列重大发展政策，明确任务，强化措施，着力推动三次产业联动发展。二是全力以赴保企业。牢固树立保企业就是保增长的发展理念，加强经济运行调度，坚持市县区领导联系重点企业制度，深入企业及时协调解决问题。出台了扶持政策，鼓励企业多产多销和扩大技改投入。全面清理和取消了一批行政事业性和经营服务性收费项目，降低企业运行成本1.4亿元。下调社保费率，缓缴困难企业社保费，发放社保补贴、就业岗位补贴，减轻企业负担2.4亿元。三是全面落实扩内需、保增长的政策措施。争取国债项目资金4.5亿元、中央和省专项补助24.2亿元、外国政府贷款2879万美元，兑现财税政策资金28亿元。全市金融机构加大信贷投放，本外币贷款余额同比增长43.6%，高于全省平均水平9.3个百分点。鑫龙电器成功上市，神剑新材料通过上市审核，长信科技等2家企业完成上市申报，奇瑞私募筹资29.8亿元，海螺集团发行中期票据30亿元，市建投发债10亿元。四是狠抓项目建设。实行市领导联系重大项目制度，加强调度，强化服务，信义玻璃一期、宝成鞋业一期、富鑫钢铁等项目建成投产，奇瑞轿车五厂、美的日用家电、华强文化科技产业园等项目建设加快，芜湖海螺三期、新兴铸管三山工业园、集瑞联合重工、玉柴联合动力、新联造船新厂、德豪润达光电等项目开工建设。核电项目前期工作取得积极进展。五是着力完善综合交通体系。充分利用国家实施扩内需的一揽子计划，上了一批我们一直想上而没有条件上的重大基础设施项目，为芜湖的长远发展奠定了坚实基础。宁安城际铁路、芜雁高速、芜申运河工程施工进展顺利，合福客运专线开工建设，青弋江分洪道项目获准立项。合芜杭客运专线、皖赣铁路新建双线、5万平方米的芜湖火车站改扩建、四条铁路既有线电气化改造、长江公路二桥前期工作全面启动。项目建成后，芜湖将成为全国重要的综合交通枢纽。六是积极扩大消费需求。认真落实家电、汽车、摩托车下乡消费政策，财政补贴3011万元，拉动了城乡消费。及时调整房地产政策，增加市场供给，增强消费信心。发放补贴1982万元，鼓励普通商品房消费，对9210户城市低收入住房困难家庭实行应保尽保。全年房地产开发完成投资210亿元，增长39.8%；销售商品房266万平方米，增长50%，价格保持基本稳定。安置房开工286万平方米、竣工111万平方米，廉租房开工43.5万平方米、竣工22.6万平方米。

（二）着力提升自主创新能力。紧紧把握省委、省政府设立合芜蚌自主创新综合配套改革试验区的机遇，全面推进创新型城市建设。一是出台了自主创新综合配套改革实施方案，财政安排7.2亿元，积极推进试验区建设。制定了“小巨人”企业培育办法，将连续五年每年安排1亿元，着力扶持一批自主创新能力较强的中小企业加速发展。实施了51个国家和省级重点科技项目，12项成果获省科技进步奖。新认定高新技术企业51家、高新技术产品80个，新增安徽省名牌产品12个。专利申请量3750件、授权量2196件，均居全省第一。高新技术产业总产值600亿元，增长30%。二是加强创新投入体系建设。设立了规模2亿元的创业（风险）投资引导基金和2.5亿元的瑞建汽车电子及关键零部件创业投资基金；成立了国富产业投资等3家创业投资公司，注册资金合计17.3亿元。三是加快创新平台建设。国家级汽车节能环保工程实验室、现代显示技术研究中心、汽车电子及关键零部件企业孵化器建设进展顺利。我市被国家确定为节能环保汽车及零部件高新技术产业基地，经济技术开发区成为国家级新型工业化示范基地。五七二О厂获国家级企业技术中心认定，新增省级工程技术研究中心8家、省级实验室3家、省级企业技术中心14家。与中国科技大学共建软件服务外包产业园，与复旦、合肥工业大学共建技术转移中心。四是大力引进人才。制定了创新人才队伍建设若干意见，强化政策措施，完善人才引进、培养和开发机制，新增中高级人才6000多人。我市成为全国科技进步先进市，繁昌县、芜湖县、弋江区成为全国科技进步先进县区。奇瑞公司成为国家国际科技合作基地、全国海外高层次人才创新创业基地、国家知识产权示范企业。

（三）大力调整优化产业结构。编制了装备制造、节能环保等8个重点产业规划，实施了一批重大工业项目。汽车及零部件、材料、电子电器三大支柱产业实现总产值1140亿元，增长21%。汽车产量突破50万辆，水泥产量超过1000万吨，均创历史新高。新兴产业迅速成长，装备制造产业产值160亿元，增长22%；电子信息、节能环保产业产值分别增长30%、50%。通过实施一批技术改造项目，传统产业加速升级。全市规模以上工业企业达1467户，比上年净增319户。制定了大力推进三产兴市战略的实施意见，确定了发展目标和任务，出台了8个政策性文件，编制了文化创意、现代物流等产业规划。商品交易博览城、果品冻品水产批发市场、联盛商业广场等一批大市场建设进展顺利，红星美凯龙家居市场、邮政物流、安得物流等项目开工建设。新建、改建“万村千乡市场工程”农家店200家。金融体系建设取得重大进展。招商、中信、光大银行芜湖分行和繁昌、芜湖县农村合作银行挂牌营业。3家保险公司、1家期货公司、1家证券公司来芜设立分支机构。全国首家中资汽车金融公司——奇瑞徽银汽车金融公司正式运营。15家小额贷款公司获批开业。新增注册资金5000万元以上的担保公司10家，新增典当公司2家。全市金融机构本外币存贷款余额分别达971.2亿元、809.9亿元，分别比年初新增241亿元、245.9亿元，有力支撑了经济社会发展。新建、改建3家四星级酒店，7家按四星级以上标准建设的酒店项目进展顺利。创建了3个4A级、2个3A级景区，全年接待游客660万人次，旅游总收入60亿元，增长20%。成功举办第二届中国国际动漫创意产业交易会、第八届中国旅游商品博览交易会等16个会展，会展经济效应进一步显现。大力发展都市型现代农业，组建农业企业行业协会8个，新增农村合作经济组织83个，新增有机食品认证14个、绿色食品标志37个、无公害农产品认证17个。认真落实强农惠农政策，发放涉农补贴资金2.7亿元。推广35万亩超级稻丰产工程取得显著成效。粮食总产达84.9万吨，连续六年增产。

（四）加快城乡大建设步伐。去年是中心城区建设力度最大的一年，市政基础设施建设投资153亿元，大建设、大发展格局初步形成。新建、续建、改扩建城市道路36条，新建、续建城市公共设施项目15个。市政务大楼建成。保兴垾综合整治一期、赭山公园西大门改造顺利完成。实施“美化、绿化、亮化”工程，完成了9条道路杆线下地，新增了420万平方米公共绿地，建设了110公里高速公路绿色长廊工程，城市主要建筑物、主干道路和出入口全面亮化。开展了市容整治年活动，拆迁改造市区棚户区40万平方米，新建、改造15个菜市场，改造老旧小区42个，拆除违法建设60万平方米，市容市貌得到较大改观。

去年是大力推进统筹城乡发展的一年，市级财政调度资金40亿元支持县区发展。编制了三县城乡总体规划及一批专业专项规划。完成了芜南路三期、省道繁昌至铜陵段、湾石路路面改建工程，建成通乡油路50公里。完成农村公路“村村通”延伸工程264公里，农村公路专项养护1328公里，改造农村危桥39座。新增公交车217台。杨家门水厂二期、南陵和三山供水工程建成投入使用。城乡电网设施进一步完善。“川气东送”芜湖项目基本落实。完成24座小型水库除险加固，解决了8.7万农村人口饮水安全问题。新建50所标准化村卫生室、5个社区卫生服务机构。国家级农村信息化试点任务全面完成。开展了“百企联百村”活动，新增省级新农村示范镇4个、示范村35个。

去年也是生态市建设力度最大的一年，节能减排和环境保护投入达37.3亿元。开发复垦整理土地9567亩，实现了耕地总量占补平衡。规范土地市场秩序，盘活存量土地1876亩，依法处置闲置土地2650亩。加大矿山资源整合力度，依法关闭小矿山72家。实施节能项目49个，年节约标准煤12万吨。天门山一期、芜湖县、繁昌县污水处理厂投入运营，朱家桥二期、城南一期和南陵县污水处理厂主体工程基本建成，滨江污水处理厂开工建设。完成垃圾焚烧热电厂改造工程，垃圾日处理能力达1100吨。实施减排项目17个，大中型项目环境评价和“三同时”执行率均保持在100%。完成造林面积6.6万亩。南陵县国家级生态示范区建设试点通过验收，九莲塘整治工程获中国人居环境范例奖。实施了“百镇千村万户生态示范工程”，整治了澛港大桥粉尘污染、市区扬尘等突出环境问题，城市环境质量进一步提升。

（五）深入推进改革开放。加大重点领域和关键环节改革力度，为科学发展营造良好的制度环境。出台了促进农村土地承包经营权流转的意见，完成了集体林权主体改革任务，新增流转耕地10.3万亩、林地5.2万亩。深化国有企业改革，妥善处理了一些历史遗留问题。积极转变政府职能，全面推进市县区政府机构改革，对市直单位科级公务员、重点部门财务科长实施交流轮岗。对11个中心镇下放经济社会管理权限，出台了加快经济技术开发区发展的决定，进一步优化发展环境。调整市与区分税制财政体制，增强了区级发展的自主性。改革财政资金管理办法，统筹调度管理市、区财政和建投公司及事业单位资金，降低融资成本，加强风险防范。开展了市属事业单位资产资金清理集中工作，撤销单位账户652个，清理资金18.9亿元。推进惠民直达工程试点，开通惠民资金管理系统。实施成品油价格税费改革，取消了政府还贷二级公路收费。实施义务教育学校绩效工资制度改革，及时落实了相关政策。深化医药分开改革，通过集中招标降低药品采购成本6000多万元，直接让利患者2100万元。我市被确定为全国公立医院改革试点城市，医药卫生体制改革全面启动。

坚持将招商引资作为经济工作的重中之重，积极承接国内外产业转移。制定了招商绩效考核办法，强化了招商引资运行调度，组建了5个市直驻点招商机构。承办了复旦大学世界校友联谊会、国际徽商精英年会，举办了11次大型投资推介活动，全年派出小分队1.2万人次，三安光电、通用集团锻冲设备、成飞集团模具等一批大项目落户芜湖，24家外资企业扩大投资，新批外商投资企业34家，与中央企业合作项目总投资1348亿元。全年实际利用外资6.3亿美元，增长10.2%；实际利用内资678亿元，增长52.7%。建成了出口加工区二期，保税物流园区正式运营。加强进出口企业孵化培育，新增进出口实绩企业60家，全市进出口总额14.2亿美元，下降30.3%，进出口急速下滑的态势得到初步遏制。港口货物吞吐量5713万吨，增长3.6%。与大连、亳州市开展了友好结对合作，友城工作得到加强。外事、侨务、对台、外宣等工作取得新进展。

（六）全面促进社会事业发展。坚持以保障和改善民生为核心，推动经济社会协调发展。全年共实施了32项民生工程，投入26.5亿元，惠及全市230万城乡居民。社会保障覆盖面不断扩大，城镇职工养老、失业、医疗、工伤、生育保险参保人数达142.8万人次，增长10%。新型农村合作医疗参加人数100.6万人，城镇居民医疗保险参保73.4万人。企业退休人员养老金月人均增加112元，被征地农民养老保障补贴月人均增加10元，失业保险金实现了市级统筹，三县失业金月人均增加80元。创新住房公积金贷款调控机制，帮助7300户职工改善了居住条件。坚持优先发展教育，市一中新校区、安徽中加学校开工建设，南陵一中新校区建成使用，新增2所民办中职学校。芜湖职业技术学院通过国家重点示范高职院校建设评估。市老年大学成为全国先进老年大学。加强公共卫生事件应急能力建设，有效防控了甲型H1N1流感、手足口病疫情。弋矶山医院、市二院、四院、中医院、城东新区医院基础设施项目建设加快，2家民营医院落户芜湖。加强公共文化服务体系建设，推进文化事业大繁荣大发展。隆重举行了纪念新中国成立和芜湖解放60周年庆祝活动，组织开展了系列文化活动演出300多场。市图书馆、文化馆、科技馆免费接待群众35万人次。建成9个镇综合文化站、90个农家书屋、16个村农民体育健身场地，新增66个自然村通广播电视。我市被评为全国文化体制改革先进地区，繁昌县被评为全国文化先进县。成功举办市第十一届运动会，鸠江区金湾社区等5个单位被评为全国群众体育先进单位。我市运动员在十一届全运会获得3金1银1铜的历史最好成绩，位居全省第一。深入开展文明创建活动和爱国卫生运动，我市在全国114个公共文明指数测评城市中位列第21位。召开了首届芜湖慈善大会，5个单位和个人获中华慈善突出贡献奖。完成了第六届社区居委会换届选举。镜湖区、藕香园社区分别被评为全国和谐社区建设示范城区、示范社区。开展了基层司法所“进万村大服务”活动。平安芜湖建设取得新成效，群众安全感满意率稳步提高。信访秩序总体向好，安全生产形势稳定。积极开展双拥创建工作，国防后备力量建设进一步加强。民族、宗教、人防、气象、档案、地方志、老龄、残疾人等事业取得新进展。

过去的一年，在加快推进经济社会发展的同时，我们更加重视政府自身建设，坚持勤政廉政，努力提高推动科学发展的能力。扎实开展了深入学习实践科学发展观活动，突出实践特色，突出解决问题，进一步增强贯彻落实科学发展观的自觉性和坚定性。自觉接受人大监督和政协民主监督，办理人大议案2件、建议114件，政协提案358件，办复率100%。加强与工会、共青团、妇联等人民团体的联系，认真听取各方面意见和建议。制定了重大行政决策程序规定，进一步规范决策行为。优化整合行政审批流程，实行 “一窗式”综合办理，不断提高审批效率。完善政府投资项目招投标制度，加大了政府信息公开力度，推动行政权力公开阳光运行。建成了市政务统一办公平台，政务信息化水平得到提升。调减市直机关公务费预算支出1026万元，厉行勤俭节约，降低行政成本。大力推行“一线工作法”，对73个单位开展政风评议，着力提高行政执行力。积极开展网络问政，组织领导干部“在线访谈”，加强与“市民心声”网友互动，提高工作透明度和公众参与度。强化行政监察和审计监督，开展了工程建设领域突出问题、党政机关和事业单位“小金库”等专项治理，惩治和预防腐败体系建设工作深入推进。

各位代表，过去一年，我们走过了不平凡的历程，经受住了严峻考验，这是中共芜湖市委统揽全局、科学决策、有效应对的结果。市人大代表充分行使职权，对重点工程、重点项目、重点工作等加强监督服务；市政协委员充分发挥自身优势，对承接产业转移、绿化美化、环境保护等全力推动支持，同舟共济，携手奋进，形成了推动发展的强大合力。全市广大干部群众倍加珍视风正气顺、团结奋斗的政治生态，始终保持奋发有为、昂扬向上的精神状态，凝心聚力，开拓创新，聚集了加速崛起的巨大力量。在这里，我谨代表市人民政府，向全体人大代表、政协委员，向全市广大工人、农民、知识分子、干部、驻芜解放军指战员、武警官兵和政法干警，向各民主党派、工商联、各人民团体、无党派人士、离退休老同志和社会各界人士，向在我市创业的投资者、建设者，向为芜湖发展作出积极贡献的中央、省直各部门和海内外友好人士，表示衷心的感谢和崇高的敬意！

各位代表，面对来之不易的成绩，我们更为清醒地看到，经济社会发展和工作中还存在着不少矛盾和问题。主要是：经济总量不够大，外向度偏低；战略性新兴产业规模较小，企业自主创新能力还不够强；农业产业化水平不高，第三产业发展相对不足；节能减排任务艰巨，资源环境与发展的矛盾还很突出；城乡居民收入增长不够快，部分群众生活还比较困难；新农村建设步伐还不快，城市规划建设管理水平有待进一步提升；政府职能转变还不到位，效能建设有待进一步加强。这些问题我们一定倍加重视，采取切实有效的措施着力加以解决。

二、2010年工作主要目标和任务

2010年是完成“十一五”规划的冲刺之年，也是谋划“十二五”发展的布局之年。我们既面临严峻挑战，更有着重大机遇。当前，中央应对危机的一揽子政策效应显现，我国经济回升向好。近年来我们大力实施六大战略，产业优势更加突出，经济基础更加坚实。国家即将启动皖江城市带承接产业转移示范区建设，明确我市为示范区核心城市、皖江开发开放的龙头城市，未来要将芜湖建设为全国重要的制造业基地、现代物流中心、创新型城市和现代化滨江组团式大城市，这将为我们扩大开放带来巨大机遇，为我们加速崛起增添强大动力。可以说，当前芜湖已经进入历史上最好的发展时期之一，具备了加快发展的内外部条件，迈入了厚积薄发、加速崛起的新阶段。同时我们也应看到，国内经济回升基础还不牢固，结构性矛盾仍很突出，经济发展内在动力仍然不足。世界经济全面复苏的不确定因素较多，国际金融危机影响依然存在，发展的复杂性不容低估。我们一定要全面辨证地看待形势，既要充分看到有利条件和积极因素，审时度势、抢抓机遇；又要对困难和挑战有足够的估计，未雨绸缪、化危为机，坚持既定目标不动摇，鼓足赶超劲头不懈怠，推进科学发展不折腾，坚定信心，锐意进取，努力开创芜湖经济社会发展新局面。

今年工作的总体要求是：以邓小平理论和“三个代表”重要思想为指导，深入贯彻落实科学发展观，紧紧围绕加快经济发展方式转变的战略任务，更加注重调整经济结构，更加注重自主创新，更加注重统筹城乡，更加注重加大有效投入，更加注重深化改革开放，更加注重保障改善民生，全面实现“十一五”规划目标，努力推动经济社会更好更快地发展。

综合考虑各种因素和条件，今年经济社会发展的主要预期目标是：全市生产总值增长14.5%；财政收入与经济同步增长；全社会固定资产投资增长30%；城镇居民人均可支配收入增长8%，农民人均纯收入增长7%；新增城镇就业岗位5万个，城镇登记失业率控制在4%以内；人口出生率控制在10‰以内；节能减排完成省政府下达任务。实现上述目标任务，必须着重抓好以下六个方面工作：

（一）以实施工业强市和三产兴市战略为主线，优化产业结构，推动经济又好又快发展

以大项目、大企业为工作的着力点，实施工业与服务业发展“双轮驱动”，推进产业体系向高端化发展，提升经济综合竞争力。

坚持走新型工业化道路，切实提高工业增长的质量和效益，到2015年，打造汽车、材料、光电三个千亿级产业，两个千亿级企业。一是发展壮大支柱产业。全力做好奇瑞公司项目服务，支持奇瑞公司创新、创业、创名牌。整合国际国内资源，引进关键零部件企业，加快推进新能源汽车、商务车项目建设。充分发挥海螺集团、新兴铸管、精诚铜业等企业技术、资金优势，积极支持企业新上一批重大项目，引进新型材料企业，做大做强特色材料产业。推进美的日用家电基地等项目建设，积极引进知名电器企业，形成产业集聚，提升发展水平，打造国内一流的家电产业基地。重点支持数控机床、起重运输设备等重大装备项目建设，迅速做大做强装备制造业。二是加快培育战略性新兴产业。全力推进三安光电、德豪润达光电、信义光伏玻璃、明远光伏电池等光电项目，推动光电产业爆发式增长，打造中国“光谷”。推进海创节能装备制造基地、杉杉生物药业科技园等一批重大项目建设，大力发展节能环保、电子信息、生物医药等产业。集中财力人力，支持各县区发挥优势，扬长避短，重点打造一、两个各具特色的战略性新兴产业。三是改造提升传统产业。引导扶持企业加大技术改造和产品研发投入，加快培育品牌，提升行业竞争力。加快新联造船新厂、宝成鞋业二期、圣罗纺织等项目建设，做大产业规模。

以金融、物流、文化创意、服务外包和区域性大市场建设为重点，着力发展现代服务业，加速推进第三产业扩张规模、优化结构，打造宁汉之间具有鲜明特色的服务业中心。加强金融体系建设，积极引进银行、证券、期货、保险机构来芜设立分支机构，组建或引进金融租赁公司、财务公司、小额贷款公司、担保公司、典当机构。大力发展一级法人金融机构，支持扬子银行做大做强，加快组建村镇银行，改制成立南陵县农村商业银行。优化金融生态环境，打造信用芜湖。推进银企合作，引导金融机构加大信贷投放。市建投和经济技术开发区建投择机发债30亿元，争取新增3户以上企业完成上市申报。大力发展现代物流业，加快建设出口加工区、芜湖港、裕溪口、城东新区等物流园区，争取启动三山临港物流园区的规划建设。加快引进现代商业业态，完善商贸流通体系。加快已开工的专业大市场建设进度，大力引进特色大市场，提升我市服务业的辐射力、带动力。推进华强文化科技产业园、鸠江区文化创意产业园、芜湖古城等项目建设，做大做强文化产业。加快滨江公园二期、马仁乌霞等景区开发，建成一批星级宾馆，整合精品旅游线路，推进与南京都市圈、长三角旅游一体化，促进旅游业大发展。举办一批特色品牌会展，着力培育会展经济。大力发展服务外包业，重点支持弋江区、经济技术开发区率先取得突破。积极发展会计、审计、咨询、评估等生产性服务业。加大房地产市场调控力度，规范房地产市场秩序，加快已出让地块开发进度，增加普通商品住房供给，鼓励居民自住和改善性住房需求，保持房地产市场健康稳定发展。加大保障性住房建设力度，开工建设安置房168万平方米、竣工280万平方米；开工建设廉租房50.7万平方米、竣工11.4万平方米，着力解决城市低收入家庭住房困难。

加快现代农业发展步伐。着力引进全国农业产业化知名大企业，加大对龙头企业支持力度，提高农产品精深加工水平，做大产业规模。认真落实强农惠农政策，努力提高农民生产积极性。推广农业标准化生产，提升超级稻和畜牧、水产等优势特色产业，积极创建省级以上农业标准化示范区和优质农产品标准化生产基地，争创国家级现代农业示范区。加快发展农民专业合作社等服务组织，加强农业科技服务，帮助农民解决生产中的困难。

（二）以自主创新和生态建设为核心，转变发展方式，提升可持续发展能力

转变经济发展方式已刻不容缓，我们必须突出提高自主创新能力、加强生态环境建设这两大战略重点，在发展中促转变，在转变中谋发展。

全面推进合芜蚌自主创新综合配套改革试验区建设。坚持有所为、有所不为，集中有限的人力、财力和物力，重点在形成科技成果、人才成果、产业成果和改革成果上取得突破。积极争取国家级、省级科技计划项目，重点支持产业优化升级、自主创新、公共服务体系建设和重大科技成果转化。着力培育高新技术产业，力争在战略性新兴产业发展上取得重大突破。实施“小巨人”企业培育工程，做大一批研发能力强、成长潜力大的“专、精、特、新”企业。强化政府服务和财税政策支持，鼓励企业建设研发机构，争取组建10家省级以上工程（技术）研究中心、重点实验室、企业技术中心，申报高新技术企业50家、高新技术产品60个。加快高新技术创业中心国家级企业孵化器、芜湖汽车电子及关键零部件企业孵化器、县区科技企业孵化器建设步伐。坚持以企业为主体、市场为导向，推进产学研结合，加强与高校院所的合作，组建产学研战略联盟，力争在科技成果产业化上取得新突破。认真做好基础工作，积极创建国家知识产权示范城市。深化人才评价和分配制度改革，建立健全创新人才引进和流动机制，支持企业设立营销中心、财务中心，吸引高端人才来芜创业发展。

大力加强生态环境建设。积极发展低碳经济，着力推进低碳技术应用，实施一批节能减排高技术项目。切实落实节能减排目标责任制，加强对重点耗能企业监控。加快滨江污水处理厂建设进程，开工建设城东污水处理厂，全面推进市区截污和雨污分流工程建设。加强重点减排项目建设，实行主要污染物减排动态监管。强化项目 “三同时”环境监察，加强规划环境影响评价管理，严禁污染类项目进入芜湖。开展环保专项行动，加大对油烟、烟尘、噪声等污染整治，加强对重点行业和环境敏感地区的污染排查，关闭所有立窑水泥生产线，及时消除环境安全隐患。加强耕地保护，深入开展闲置土地专项清理，强化土地市场动态监管。以土地投资强度、单位产出率等为重点，加强指标考核，促进集约节约用地。加强矿产资源开发整合，促进资源合理利用。开展植树造林，加快国土绿化进程，增加森林碳汇，努力使芜湖的山更绿、水更清、天更蓝。

（三）以深化改革和扩大开放为动力，完善体制机制，增强经济社会发展活力

积极推进重要领域和关键环节改革，努力解决当前经济社会发展中的深层次矛盾。一是深化统筹城乡综合配套改革。抓好南陵大浦试点，推进土地使用、农村金融、财政体制、户籍制度等改革，探索城乡一体化发展新路。完成全市农村集体土地、房屋确权发证工作，深化集体林权制度配套改革。鼓励土地承包经营权流转，支持龙头企业等市场主体集约化、规模化经营。加强农村基本公共服务体系建设，健全为民服务全程代理制。完善征地补偿安置政策，切实解决被征地农民就业、住房和社会保障问题。二是深化行政管理体制改革。优化政府部门职能配置。深化镇级机构改革和村级综合改革，整合镇级行政服务资源，推进“撤村建居”工作。三是深化企事业单位改革。完善国企考核机制，做好国企改革后续工作。按照中央和省里统一部署，分步实施事业单位绩效工资制度改革。四是深化医药卫生体制改革。扎实推进公立医院改革全国试点工作，探索建立以三大医疗集团为核心的法人治理结构。加快推进基本医疗保障制度建设，全面执行国家基本药物制度，健全基层医疗卫生服务体系。全面实施省基层医药卫生体制综合改革试点，促进基本公共卫生服务均等化。五是积极发展民营经济。弘扬创新文化，营造全社会尊重、爱护、宽容、保护企业家的良好氛围，让一切创造财富的源泉充分涌流。深入推进创业富民战略，完善政策支撑体系，加大对民营企业支持力度，积极帮助企业解决资金、技术、信息等方面的困难。

大力发展开放型经济，努力形成多层次、宽领域、全方位的大开放格局。一是全面推进皖江城市带承接产业转移示范区建设。编制示范区建设实施方案，建立健全示范区工作推进机制。加快保税港区规划、建设和申报审批工作，强化规划引导，加大基础设施投入，加快建设步伐。二是掀起招商引资新高潮。加大利用外资力度，提高利用外资的质量和水平。充分利用小分队招商、驻点招商、代理招商、产业招商等有效方式，加大项目跟踪落实力度，推进与中央企业的合作，力争一批重大项目落地。推动经济技术开发区“二次创业”，力争引进大企业、大项目取得新成效。全面启动长江大桥综合经济开发区新区规划、建设，力争形成承接产业转移新高地。扎实推进芜湖高新区优化升级，力争高新技术产业发展形成新突破。着力改善各省级开发区基础条件，完善招商服务机制，力争新兴产业招商取得新进展。三是加强对外合作。积极参与泛长三角发展分工与合作，重点在市场准入、信息平台、旅游营销、社保体系等方面取得突破。主动加强与大连、亳州市的务实合作，积极发展与国内外城市的友好关系。支持优势企业以资本为纽带，开展多种形式跨区域联合重组，加快国际化、集团化发展步伐。四是稳定发展对外贸易。实施市场多元化战略，大力引进外向型企业，做好进出口企业培育孵化工作，提高出口产品档次、附加值、竞争力，扩大高技术装备进口，实现外贸保份额、调结构、促平衡。

（四）以城市大建设和新农村建设为抓手，统筹城乡发展，加快城市化进程

坚持“三个集中、六个一体化、两个延伸”的发展方向，研究制定推进城市化的指导意见和政策措施，努力把芜湖打造成宜业、宜居、宜游城市。

着力加强城乡规划管理。按照经济社会发展规划、城市总体规划、土地利用规划、产业布局规划“四规合一”的要求，优化空间组团布局，强化规划法律约束。编制三县四区发展战略规划，加快实施新一轮城市总体规划，编制芜湖火车站新站、“两江两湖”等概念性规划，积极构建全市域、多层次、更科学的规划体系。

继续加大政府投入，进一步拉开城市框架，推进城市“东扩、南进、中提升”。加快开发城东新区，高起点规划，大手笔建设，着力打造生态和谐的现代化城市示范区。完善城南基础设施，推进“两江两湖”规划建设，着力打造山水秀美的高新产业集聚区。加大老城区改造力度，提高畅通能力，提升城市品位，着力打造环境优越的现代服务业核心区。发展立体交通，优化城区路网体系。建成赤铸山东路延伸段、三山区疏港大道、长江路南延伸高架等道路；开工建设九华山路高架、三环路南延伸段、滨江南路南延伸段等道路工程。建成三环路跨青弋江大桥、袁泽桥西幅桥。推进城市交通枢纽建设，加快铁路项目、芜雁高速公路、芜申运河建设进度，完成长江公路二桥前期工作。推进城市环境整治，完成神山公园、中央公园、扁担河景观、环赭山改造工程，继续加强保兴垾综合治理，启动板城垾、大洋垾整治工程。实施绿化、美化、亮化工程，新增绿化面积480万平方米、亮化街道15条。强化区级责任，加快区属道路、景观、住宅小区整治等项目建设。完成市区棚户区改造40万平方米。开展市容整治巩固年活动，推进数字城管系统开发应用，实施城市路名规划，提高城市管理信息化、规范化水平。加大查处违法建设工作力度，完善长效监管机制。创新物业管理机制，提高物业服务水平。大力发展公共交通，加强出租车、公交车、客运车管理，改善公共交通服务。

着力推进新农村建设。抢抓国家推进小城镇建设历史机遇，加快三县县城、中心镇建设步伐，提升规划建设管理水平。以村庄整体整治为重点和突破口，积极实施城镇建设用地增加与农村建设用地减少挂钩项目，优化农村土地利用布局，形成居住相对集中、产业集聚发展的新农村建设发展新格局。加强农村基础设施建设，完成100公里农村公路“村村通”延伸工程，解决12万农村人口饮水安全问题；加大重点水利工程兴修力度，提高农业综合生产和防汛抗旱能力；开工建设外龙窝湖综合整治工程，完成水阳江、青弋江流域芜湖段水利血防工程。深入开展爱国卫生运动，推进农村生态环境综合整治，从今年开始，五年内完成全市农村主要沟渠湖塘清淤疏浚，加快改水、改院、改厕、改圈，启动垃圾处理城乡一体化试点，治理白色垃圾污染，创建一批国家级、省级环境优美镇和生态村，争创一批省级新农村示范村。

（五）以保障改善民生和构建和谐社会为目标，坚持以人为本，全面推进社会建设

坚持把保障和改善民生作为政府工作的出发点和落脚点，把顺民意、解民忧、厚民生的事情办实办好。安排投入28.5亿元，实施38项民生工程。在去年实施的32项民生工程基础上，增加6项民生工程，确保人民群众生活得到更大的改善。

发展教育、文化、卫生事业。推进中小学标准化建设，促进义务教育均衡发展。健全学生资助制度，保障经济困难家庭、进城务工人员子女平等接受义务教育。发展职业教育，建成芜湖工业学校、芜湖师范学校新校区主体工程。加快普及高中阶段教育进程，建成市一中新校区、安徽中加学校。支持在芜高校发展，提高高等教育质量。深化教育改革，加强学校管理，提高教育现代化水平。鼓励民间资本兴办教育机构，扩大民办教育规模。加强文明创建工作，倡导文明风气，提高市民素质，净化社会环境，争创全国文明城市。加快市艺术剧院建设，建成4个镇文化站、79个农家书屋。开展文化惠民活动，建设文化示范街道、示范社区，丰富群众精神文化生活。推行县区文化行政综合执法，规范文化市场管理。开展全民健身运动，提升竞技体育水平，力争省运会比赛取得好成绩。开工建设市三院突发传染病救治中心，加快城东医院建设，建成弋矶山医院、市二院、市中医院、市四院改扩建项目。坚持中西医并重，促进中医药事业发展。健全工作制度，确保食品药品安全。稳定低生育水平，提高出生人口素质，加强流动人口计生服务管理和出生人口性别比综合治理。

加强就业和社会保障工作。推进城乡统筹就业全国试点、国家级创业型城市试点，完善就业服务体系，实施创业扶持工程，以创业带动就业。落实小额担保贷款、就业援助等政策，重点扶持高校毕业生、返乡农民工和困难群体就业。建成芜湖技师学院新校区一期工程，实施新技师培养计划。实行工伤保险、城镇居民医疗保险市级统筹，开展城镇居民医疗保险门诊统筹试点。完善城乡居民最低生活保障和农村五保供养制度，实现应保尽保。发展社会福利和慈善事业，建立健全养老服务体系。加强住房公积金使用管理，扩大制度覆盖面。

提高社会管理水平。启动农村社区建设工作，创建一批村务公开、民主管理示范村镇。开展和谐社区示范创建活动，推动社区信息化建设。开展第六次全国人口普查。积极支持驻芜部队建设，做好国防动员、民兵预备役工作。推进法治城市、法治县区创建活动，完成“五五”普法宣传和依法治理工作目标。做好妇女儿童发展规划的评估、编制工作。加强信访工作，严格责任追究制，强化社情研判和矛盾纠纷排查调处，有效预防和妥善处置事件，维护群众合法权益。落实安全生产责任，加强安全生产基层基础工作，开展非煤矿山等重点行业隐患排查治理。推进平安芜湖建设，强化社会治安综合治理，加强镇街（综治）中心建设，健全应急管理体系，严厉打击犯罪行为，维护国家安全和社会和谐稳定。

（六）以勤奋务实和反腐倡廉为重点，提高行政效能，打造服务型政府

2010年是应对国际金融危机的关键之年，各种困难和挑战很多，保证完成今年各项任务，必须更加勤奋努力，更加廉洁自律。

坚持加强学习，增强发展本领。着力建设学习型机关，树立崇尚学习的良好风气，不断用新理论新知识武装头脑，提高推进科学发展的本领。弘扬勤奋刻苦、恪尽职守、甘于奉献的敬业精神，倡导时时在状态、人人有激情、事事争先进的进取精神，努力增强工作的主动性、前瞻性、创造性。正确把握宏观发展环境和趋势，创新理念和方法，切实做好“十二五”规划编制工作，努力增强规划的科学性、预见性、指导性。

坚持转变作风，提升行政效能。大兴调查研究之风，深入基层，重心下移，切实做到在一线发现问题，在一线解决问题。坚持密切联系群众，了解群众疾苦，倾听群众呼声，帮助群众解决困难。加强电子政务建设，推行网上办公，精简会议、文件。完善目标管理体系，建立科学考核评价机制。改进政风评议，加大服务对象评价的权重。完善行政问责制度，坚决治懒治庸。强化政务督办，做到各项工作有部署、有督促、有检查、有落实。

坚持依法行政，规范施政行为。自觉接受人大监督和政协民主监督，认真办理人大议案、建议和政协建议案、提案，充分听取各民主党派、工商联、无党派人士及各人民团体的意见，强化监察、审计等专门监督，高度重视人民群众监督和新闻舆论监督，不断改进政府工作。完善行政审批“快捷通道”和并联审批机制，加快推进网上审批建设，实行电子监察、限时办理，提高审批效率。规范行政事业性收费和服务性收费，加强对各项收费和市场价格的监管。深入推进政务公开，开展网络问政，加强与市民和网友的直接交流，完善新闻发布制度，提高政府工作透明度。严格落实行政执法责任制和执法过错责任追究制，规范行政行为。

坚持清正廉洁，筑牢拒腐防线。全面落实廉政工作责任制，扎实推进惩治和预防腐败体系建设。加强廉政教育和廉政文化建设，巩固和完善用人、用钱、用地、用权制度，以制度管人管事管权。加强对权力运行的监督制约，对所有经营性用地开发项目、政府性投资项目实行第三方审计制度和政府审计复查制度，防止权力失控、行为失范。坚持勤俭办事，大力压缩公用经费开支，把有限的资金和资源更多地用在发展经济和改善民生上。

各位代表，芜湖正处在加速崛起的关键时期，江城大地激情奔涌，江城儿女豪情满怀。让我们在中共芜湖市委的坚强领导下，万众一心，勇往直前，为实现“十一五”经济社会发展目标、夺取全面建设小康社会新胜利而努力奋斗！
